# Clinical characteristics and beta cell function in Chinese patients with newly diagnosed type 2 diabetes mellitus with different levels of serum triglyceride

**DOI:** 10.1186/s12902-015-0018-1

**Published:** 2015-04-29

**Authors:** Shuang Zheng, Huan Zhou, Tingting Han, Yangxue Li, Yao Zhang, Wei Liu, Yaomin Hu

**Affiliations:** Department of Endocrinology, Renji Hospital, School of Medicine, Shanghai Jiaotong University, NO.160, Pujian Road, Shanghai, 200127 China

**Keywords:** Newly diagnosed type 2 diabetes, Beta cell function, Insulin resistance, Triglyceride

## Abstract

**Background:**

To explore clinical characteristics and beta cell function in Chinese patients with newly diagnosed drug naive type 2 diabetes mellitus (T2DM) with different levels of serum triglyceride (TG).

**Methods:**

Patients with newly diagnosed T2DM (n = 624) were enrolled and divided into different groups according to levels of serum TG. All patients underwent oral glucose tolerance tests and insulin releasing tests. Demographic data, lipid profiles, glucose levels, and insulin profiles were compared between different groups. Basic insulin secretion function index (homeostasis model assessment for beta cell function index, HOMA-β), modified beta cell function index (MBCI), glucose disposition indices (DI), and early insulin secretion function index (insulinogenic index, IGI) were used to evaluate the beta cell function.

**Results:**

Patients of newly diagnosed T2DM with hypertriglyceridemia were younger, fatter and had worse lipid profiles, glucose profiles, and high insulin levels than those with normal TG. There is no difference in early phase insulin secretion among groups of newly diagnosed T2DM patients with different TG levels. The basal beta cell function (HOMA-β and MBCI) initially increased along rising TG levels and then decreased as the TG levels rose further. The insulin sensitivity was relatively high in patients with a low level of TG and low with a high level of TG.

**Conclusions:**

Hypertriglyceridemia influences clinical characteristics and β cell function of Chinese patients with newly diagnosed T2DM. A better management of dyslipidemia may, to some extent, reduce the effect of lipotoxicity, thereby improving glucose homeostasis in patients with newly diagnosed T2DM.

**Electronic supplementary material:**

The online version of this article (doi:10.1186/s12902-015-0018-1) contains supplementary material, which is available to authorized users.

## Background

Type 2 diabetes mellitus (T2DM), a chronic metabolic disease, has become a serious issue in China with increasing incidences over the past decades. Insulin resistance and impaired insulin secretion are considered as primary pathophysiological factors in the development of T2DM [1]. The UK Prospective Diabetes Study has shown that the β cells function begins deteriorating before T2DM diagnosis and often years before the patients presenting clinical symptoms [2,3]. In most patients with T2DM, the metabolic control and β cell function progressively deteriorate as the duration of diabetes increases [4,5]. Because the β cell function declines with diabetic duration, patients with newly diagnosed drug naive T2DM would be suitable subjects of T2DM study. Hyperlipidemia, an increase of total cholesterol (TC) and/or triglycerides (TGs) in the serum, is one of the most common T2DM related comorbidities [6]. Diabetic dyslipidemia is characterized by moderately increased TG levels and reduced high density lipoprotein-cholesterol (HDL-C), which is a well-recognized causes for atherosclerotic cardiovascular diseases [7,8]. In our previous studies, we found that the mice with heterozygous lipoprotein lipase (LPL) deficiency with a rising serum TG display a disorder of glucose metabolism and deterioration of β cells function [9]. Therefore, it is important to investigate the role of lipid profiles in patients with newly diagnosed T2DM and whether the different TG levels influence the glucose control and insulin secretion. Several studies have reported the characteristics of newly diagnosed T2DM; however, very few focused on the relationship between TG and newly diagnosed T2DM. We hypothesized that newly diagnosed T2DM patients with high serum TG level might have worse clinical characteristics and more deteriorated β cells function than patients with normal TG level. Hence, in the present study, we examined the clinical characteristics and β cells function in patients with newly diagnosed drug naive T2DM with different levels of TG.

## Methods

### Patients

Patients with newly diagnosed T2DM (n = 624) were enrolled in the study between January 2008 and December 2009 in Renji Hospital, School of Medicine, Shanghai Jiaotong University, Shanghai, China. All patients had been diagnosed with T2DM within 5 months before the study enrollment and none of them have received drug treatment for T2DM. The diagnosis of T2DM was based on WHO diagnostic criteria established in 1998 [10]. The patients were firstly divided into normal serum TG(newly diagnosed T2DM with TG up to 1.70 mmol/L, n = 348)and high serum TG (newly diagnosed T2DM with TG over 1.70 mmol/L, n = 276)and further divided into four sub-groups according to the quartile of serum TG (Group 1: newly diagnosed T2DM with TG up to 1.13 mmol/L, n = 152; Group 2: newly diagnosed T2DM with TG of 1.14 to 1.56 mmol/L, n = 158; Group 3: newly diagnosed T2DM with TG of 1.57 to 2.27 mmol/L, n = 158; and Group 4: newly diagnosed T2DM with TG of 2.28 to 11.65 mmol/L, n = 156). The study was carried out in compliance with the declaration of Helsinki. The study protocol was approved by the Ethical Committee of Renji Hospital, School of Medicine, Shanghai Jiaotong University, Shanghai, China (Number: Renjikls N026). Written informed consent for participation in the study was obtained from participants.

### Data collection

The patients demographic data and clinical data were collected including age, sex, body height, weight, waist circumference (WC), systolic blood pressure (SBP), diastolic blood pressure (DBP), and levels of TG, TC, HDL-C, low density lipoprotein-cholesterol (LDL-C), fasting plasma glucose (FPG), 2 h postprandial glucose (2hPG), serum insulin concentrations at different time point, and hemoglobin A1c (HbA1c). Blood pressure was measured after 30 minutes of rest. The body mass index (BMI) was calculated as weight (kg) divided by the square of height (m^2^). Plasma lipid profiles were determined using fully automatic biochemistry analyzer (Hitachi 7020, Hitachi Co., Tokyo, Japan). A standard oral glucose tolerance test (75 g glucose load) and insulin releasing tests were performed after a 10-hour overnight fast. Plasma samples were obtained at 0, 30, 60, 120, and 180 minutes to measure glucose (Hitachi 7600–110, Hitachi Co.) and insulin (immunoradiometric assay kit, Dainabot, Tokyo, Japan) concentrations. The HbA1c levels were measured using a high-performance liquid chromatography.

The parameters including basic insulin secretion function index (homeostasis model assessment for β cell function index, HOMA-β), modified beta cell function index (MBCI), glucose disposition indices (DI), and early insulin secretion function index (insulinogenic index, IGI) were used to evaluate β cell function. Insulin sensitivity was estimated by homeostasis model assessment for insulin resistance (HOMA-IR) and insulin action index (IAI). The respective formulas for calculating the above mentioned parameters were as follows: HOMA-β = 20× I_0_/(G_0_-3.5); MBCI = I_0_ × G_0_/(G_120_ + G_60_-7); DI = IGI/HOMA-IR; IGI = △I_30_/△G_30_; HOMA-IR = I_0_× G_0_/22.5; and IAI = 1 / (I_0_ × G_0_), wherein, I_0_ (μU/mL) denotes fasting plasma insulin, I_30_ insulin level at 30 minutes after glucose load, G_0_(mmol/L) fasting plasma glucose, G_30_ plasma glucose level at 30 minutes after glucose load, G_60_ plasma glucose level at 60 minutes after glucose load, and G_120_ plasma glucose level at 120 minutes after glucose load.

### Statistical analysis

Normality was tested using the one-sample Kolmogorov-Smirnov criterion. Non-normally distributed data were log transformed before analysis. Data were expressed as mean ± standard deviation for normally distributed variables and as median (Interquartile range was 25-75%) for skewed variables. Independent-samples *t* test and one-way analysis of variance test were used for normality distributed data. Mann–Whitney *U* test and Kruskal-Wallis H test were used for non-normal distributed data and alpha level was adjusted to reduce the error risk. A P value of <0.05 was considered statistically significant. All statistical analyses were performed using the SPSS statistical package (Version 17.0, SPSS Inc., USA)

## Results

### The comparison of the demographic and basic data among patients of newly diagnosed T2DM with normal and high levels of serum TG

Clinical data and baseline characteristics of patients with normal and high serum TG are presented in Table 1. The male/female ratio had no significant difference among two groups (46.84% and 51.81% for normal TG and high TG groups, respectively; P = 0.274). Patients of newly diagnosed T2DM with high TG levels were younger than patients with normal TG level (55.98 ± 11.78 and 53.64 ± 12.04 years for normal TG and high TG Groups, respectively; P < 0.05) and their diastolic blood pressure (high TG Group) was higher than that in patients with normal TG levels (81.36 ± 10.72 and 83.83 ± 9.10 mmHg for normal TG and high TG groups, respectively; P < 0.05); however, no significant difference in systolic blood pressure was found between two groups. Compared to patients with normal serum TG levels, patients of newly diagnosed T2DM with high levels of TG were much fatter with higher BMI and WC (BMI: 24.60 ± 3.85 and 25.94 ± 4.20 kg/m^2^ for normal and high TG groups, respectively; P < 0.05; WC: 86.42 ± 9.31 and 90.06 ± 9.66 cm for normal and high TG groups, respectively; P < 0.05). These data indicate that the patients of newly diagnosed T2DM with higher level of TG were younger and fatter than patients with normal TG levels.

**Table 1:** **Demographic and clinical data of newly diagnosed T2DM patients with normal and high levels of triglyceride**

	**Normal TG Group**	**High TG Group**
	**(TG ≤1.70 mmol/L)**	**(TG >1.70 mmol/L)**
	**(N = 348)**	**(N = 276)**
Male/Female	163/185	143/133
Age (years)	55.98 ± 11.78	53.64 ± 12.04*
BMI (kg/m^2^)	24.60 ± 3.85	25.94 ± 4.20*
WC (cm)	86.42 ± 9.31	90.06 ± 9.66*
SBP (mmHg)	131.45 ± 17.95	132.82 ± 15.62
DBP (mmHg)	81.36 ± 10.72	83.83 ± 9.10*
Lipid profiles		
TG (mmol/L)	1.17 ± 0.32	2.81 ± 1.33*
TC (mmol/L)	4.85 ± 0.93	5.42 ± 1.19*
HDL-C (mmol/L)	1.42 ± 0.39	1.20 ± 0.28*
LDL-C (mmol/L)	3.04 ± 0.74	3.41 ± 0.88*
Glucose profiles		
FPG (mmol/L)	6.80 (6.30, 7.90)	7.20 (6.20, 8.52)
2hPG (mmol/L)	13.96 ± 3.55	14.57 ± 3.84*
HbA1c (%)	6.60 (6.00, 7.60)	7.00 (6.30, 8.60)*
HbA1c (mmol/mol)	48.63 (42.08, 59.56)	53.01 (45.36, 70.49)*
Insulin secretion		
INS0 (uIU/mL)	10.74 ± 8.20	11.79 ± 7.79
INS30 (uIU/mL)	27.07 ± 23.26	32.34 ± 28.32*
INS60 (uIU/mL)	41.56 ± 31.06	48.50 ± 36.98*
INS120 (uIU/mL)	49.68 ± 32.57	58.57 ± 41.25*
INS180 (uIU/mL)	33.00 ± 23.93	37.59 ± 27.42*

Data were expressed as mean ± standard deviation for normal distribution and as median (Interquartile range 25-75%) for skewed variables.

BMI: body mass index; WC: waist circumference; SBP: systolic blood pressure; DBP: diastolic blood pressure; TG: triglyceride; TC: total cholesterol; HDL-C: high density lipoprotein-cholesterol; LDL-C: low density lipoprotein-cholesterol; FPG: fasting plasma glucose; 2hPG: 2 h postprandial glucose; HbA1c: hemoglobin A1c; INS0: fasting insulin; INS30: 30 minutes postprandial serum insulin; INS60: 60 minutes postprandial serum insulin; INS120: 120 minutes postprandial serum insulin; INS180: 180 minutes postprandial serum insulin;

Group was defined by the level of serum TG. Normal TG Group: newly diagnosed T2DM with TG up to 1.70 mmol/L; High TG Group: newly diagnosed T2DM with TG over 1.70 mmol/L;

^*^P < 0.05 versus Group 1.

### The comparison of lipid profiles among patients of newly diagnosed T2DM with normal and high levels of serum TG

We compared levels of TC, LDL-C and HDL-C between patients of newly diagnosed T2DM with normal and high TG levels (Table 1). Compared to patients of newly diagnosed T2DM with normal TG levels, patients of newly diagnosed T2DM with high level of TG had higher levels of TC (4.85 ± 0.93 and 5.42 ± 1.19 mmol/L for normal and high TG groups, respectively; P < 0.05), LDL-C (3.04 ± 0.74 and 3.41 ± 0.88 mmol/L for normal and high TG groups, respectively; P < 0.05) and lower level of HDL-C (1.42 ± 0.39 and 1.20 ± 0.28 mmol/L for normal and high TG groups, respectively; P < 0.05). We concluded that the patients of newly diagnosed T2DM with high level of TG had worse lipid profiles than the patients with normal TG level.

### The comparison of glucose and insulin profiles among patients of newly diagnosed T2DM with normal and high levels of serum TG

**Figure 1:**
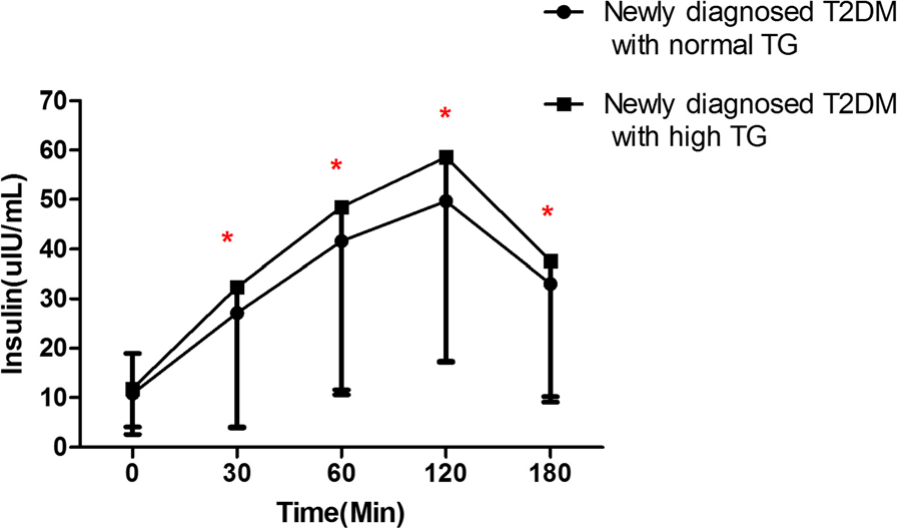
Serum insulin concentration curves in insulin releasing tests. These tests were done in patients of newly diagnosed T2DM with normal and high serum triglyceride levels. Normal TG means serum TG concentration ≤ 1.70 mmol/L and high TG means >1.70 mmol/L, * means P < 0.05.

Compared to patients with normal TG levels, patients with high TG levels had the higher levels of 2hPG and HbA1c (P < 0.05). We also performed insulin releasing tests by measuring serum insulin concentrations at various time points after patients were given 75 g glucose orally (Figure 1). We found that serum insulin concentrations after glucose load were significantly higher in patients with high TG than in patients with normal TG, suggesting that patients of newly diagnosed T2DM with high serum TG may have developed insulin resistance. These data indicate that patients of newly diagnosed T2DM with high level of TG have a worse glucose profiles and higher level of insulin.

### The comparison of β cell function and insulin sensitivity among patients of newly diagnosed T2DM with different levels of serum TG

**Figure 2:**
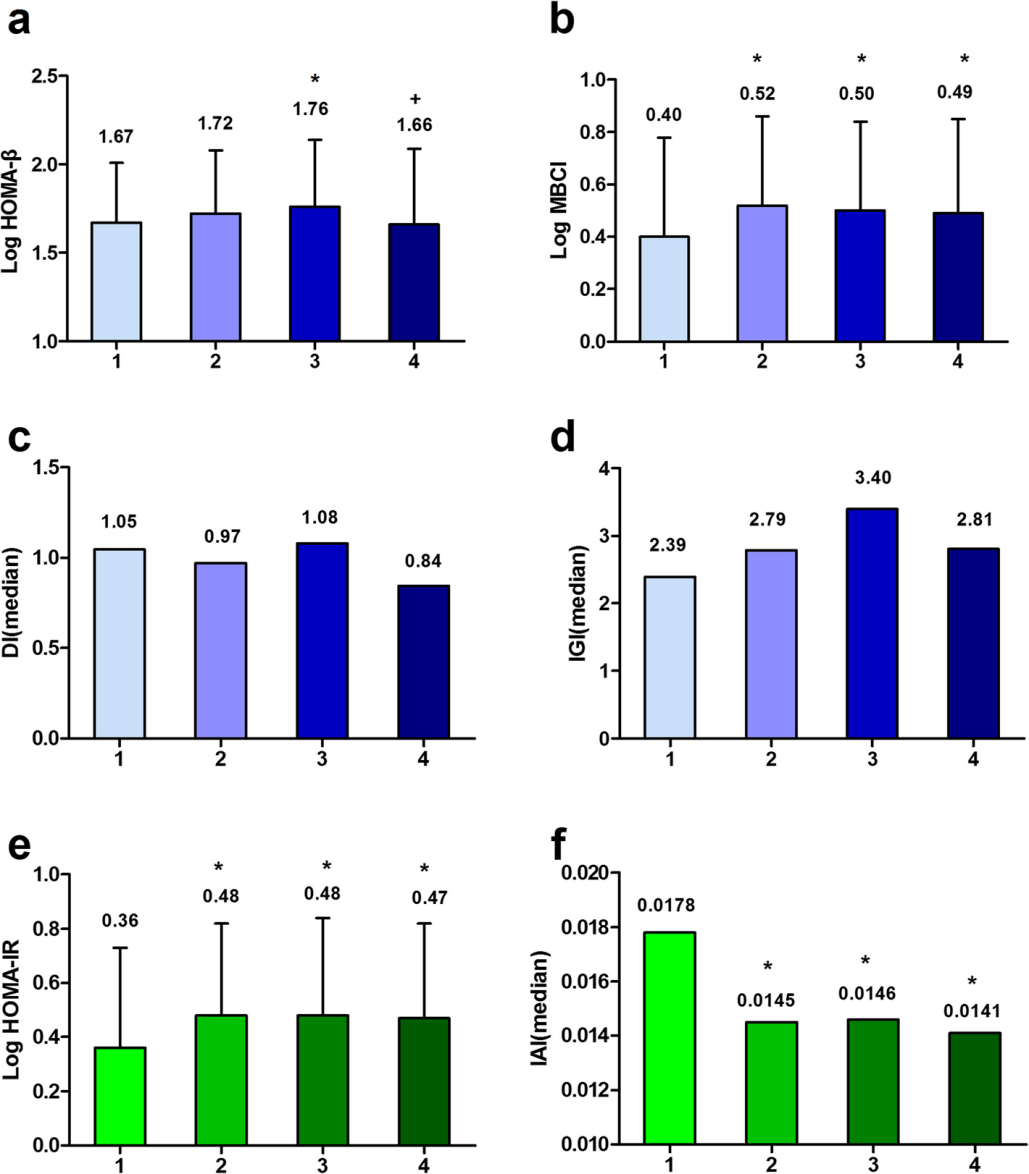
β cell function and insulin sensitivity. **(a)**: Log HOMA-β (homeostasis model assessment of β cell function) level in patients of newly diagnosed type 2 diabetes mellitus (T2DM) with different level of triglyceride (TG); **(b)**: Log modified beta cell function index (MBCI) level in patients of newly diagnosed T2DM with different level of TG; **(c)**: Glucose disposition indices (DI) level in patients of newly diagnosed T2DM with different level of TG; **(d)**: Early insulin secretion function index (IGI) level in patients of newly diagnosed T2DM with different level of TG; **(e)**: Log HOMA-IR (homeostasis model assessment of insulin resistance) level in patients of newly diagnosed T2DM with different level of TG; **(f)**: Insulin action index (IAI) level in patients of newly diagnosed T2DM with different level of TG. Group was defined by the quartile of serum TG. Group 1: newly diagnosed T2DM with TG up to 1.13 mmol/L; Group 2: newly diagnosed T2DM with TG of 1.14 to 1.56 mmol/L; Group 3: newly diagnosed T2DM with TG of 1.57 to 2.27 mmol/L; and Group 4: newly diagnosed T2DM with TG of 2.28 to 11.65 mmol/L. *P < 0.05 versus Group 1; ^#^P < 0.05 versus Group 2; ^+^P < 0.05 versus Group.

To evaluate relationship between β cell function, insulin sensitivity and serum TG levels, we divided patients into four groups according to the quartile of serum TG levels (details in *Method*, results showed in Table 2). Compared with patients in Groups 1 and 4, patients in Group 3 had the highest HOMA-β level (1.76 ± 0.38, P < 0.05), which, however, was not significantly different from that found in Group 2 patients (Figure 2a). Patients from Group 1 had the lowest level of MBCI (P < 0.05). However, there were no significant differences in MBCI levels among other groups (Figure 2b). No significant differences in IGI and DI levels were found among four groups (Figure 2c,d). The HOMA-IR and IAI were used to estimate the insulin sensitivity. Compared with Group 1, the HOMA-IR levels were elevated in Groups 2, 3, and 4 (P < 0.05); however, no significant differences were found among Groups 2, 3, and 4 (Figure 2e). In contrast, the IAI levels were significantly lower in Groups 2, 3, and 4 than in Group 1 (P < 0.05). Again, no significant differences in IAI levels were found among Groups 2, 3, and 4 (Figure 2f). In summary, there are no differences in early phase insulin secretion among patients of newly diagnosed T2DM with different levels of serum TG. The basal β cell function (HOMA-β and MBCI) increased initially along with the rising of TG levels and then decreased as the serum TG level rose further. Insulin sensitivities were relatively high in patients with low level of TG and low in patients with high level of TG.

**Table 2:** **β Cell function and Insulin Sensitivity of newly diagnosed T2DM with different levels of TG**

	**Group1 (0-25%)**	**Group2 (25%-50%)**	**Group3 (50%-75%)**	**Group4 (75%-100%)**
**β Cell Function**				
HOMA-β	47.83 (32.62, 71.70)	55.03 (35.28, 86.35)	66.14 (36.47, 95.63)*	52.39 (31.42, 82.52)^+^
MBCI	2.76 (1.67, 3.77)	3.33 (2.13, 5.26)*	3.19 (2.38, 5.46)*	3.59 (2.32, 5.13)*
IGI	2.39 (1.14, 4.78)	2.79 (0.93, 5.60)	3.40 (1.09, 8.14)	2.81 (0.82, 6.02)
DI	1.05 (0.44, 1.96)	0.97 (0.32, 1.94)	1.08 (0.32, 2.49)	0.84 (0.25, 1.83)
**Insulin Sensitivity**				
HOMA-IR	2.49 (1.70, 3.59)	3.06 (2.10, 4.62)*	3.05 (2.22, 4.86)*	3.15 (2.41, 4.87)*
IAI	0.0178 (0.012, 0.026)	0.0145 (0.010, 0021)*	0.0146 (0.010, 0.020)*	0.0141 (0.010, 0.019)*

Data were expressed as median (Interquartile range 25-75%) for skewed variables.

HOMA-β: homeostasis model assessment of β cell function; MBCI: modified beta cell function index; IGI: early insulin secretion function index; DI: glucose disposition indices; HOMA-IR: homeostasis model assessment of insulin resistance; IAI: insulin action index; T2DM: type 2 diabetes mellitus.

Group was defined by the quartile of serum TG. Group 1: newly diagnosed T2DM with TG up to 1.13 mmol/L; Group 2: newly diagnosed T2DM with TG of 1.14 to 1.56 mmol/L; Group 3: newly diagnosed T2DM with TG of 1.57 to 2.27 mmol/L; and Group 4: newly diagnosed T2DM with TG of 2.28 to 11.65 mmol/L.

*P < 0.05 versus Group 1; ^#^P < 0.05 versus Group 2; ^+^P < 0.05 versus Group 3.

## Discussion

Patients with T2DM are common to have lipid disorders. In the present study, we found that patients of newly diagnosed T2DM with higher level of TG were younger and fatter. These patients had worse lipid profiles and glucose profiles than did patients with normal TG levels. Moreover, the basal β cell function initially increased along with rising TG levels and then decreased as the TG levels rose further. Insulin sensitivities were relatively high in patients with low TG levels and low in patients with higher TG levels.

Type 2 diabetes, a heterogeneous disorder characterized by impaired insulin secretion and insulin resistance, is closely related to obesity [11]. BMI and WC usually serve as parameters to estimate the general and abdominal fat masses [12]. Several studies have reported that BMI was positively associated with an endogenous insulin secretion as assessed by serum C-peptide response in T2DM [13,14]. In addition, BMI has been suggested to be positively associated with the decreased insulin sensitivity in T2DM [15]. Although the BMI represents the degree of overweight and obesity, it fails to represent the body fat distribution. Visceral fat tissue is metabolically more active than non-visceral fat and secretes more hormones and cytokines, which promotes the development of insulin resistance and T2DM [16]. WC measurement is a simple means assessing the levels of visceral fat. Increased WC is closely associated with the increased risk of diabetes [17].

The findings in our present study are consistent with previous reports. We observed that the TG levels rise in patients as BMI and WC increase. Similarly, MBCI and HOMA-IR increase along with raising TG levels, which suggest that BMI and WC were positivity associated with insulin secretion and negativity associated with insulin sensitivity.

Several studies have demonstrated that the abnormalities in lipid profiles of patients with diabetes such as decreased levels of HDL-C, increased levels of the TC, TG, LDL-C, and very-low-density lipoprotein (VLDL)-C are associated with high insulin resistance [7,18]. Some mechanisms whereby insulin resistance could cause an alteration in lipid metabolism have been described [19-21]. Hyperinsulinemia is known to enhance the hepatic VLDL synthesis and thus may directly contribute to increased plasma TG and LDL-C levels [19]. Resistance to the action of insulin on lipoprotein lipase in peripheral tissues may also contribute to the elevated TG and LDL-C levels [21]. It has been suggested that insulin resistance may be responsible for low levels of HDL-C observed in patients with T2DM. Moreover, lipotoxicity could be one of the most important causes for T2DM. Hermans et al. [22] reported that T2DM patients with a high ratio of log (TG)/HDL-C, used to evaluate the plasma atherogenic index, tend to have a high loss rate of insulin secretion and β cell function. Therefore, a lower ratio of log (TG)/HDL-C could be beneficial to glucose control. Imamura et al. [23] have indicated that higher levels of TG (≥1.69 mmol/L) and FPG (5.5–7.0 mmol/L) are associated with a higher risk of DM preceded predominantly by β cell dysfunction. This may be attributed to that excessive plasma TG lead to elevated levels of circulating free fatty acids (FFAs) which causes the impairment of the β cell function. In our previous clinical studies, we found increased plasma triglyceride and free fatty acid levels are frequently associated with T2DM. LPL gene mutations contribute to the hypertriglyceridemia observed in T2DM patients [24,25]. In our animal experiments, we found LPL (+/−) mice with high serum TG level had worse glucose homeostasis and more serious insulin resistance in many insulin target tissues (liver, muscle, subcutaneous fat and ventral fat) than wild type mice with normal TG level[9]. In agreement, our data showed that the patients of newly diagnosed T2DM with higher levels of TG had higher levels of TC and LDL-C and lower levels of HDL-C. In addition, HOMA-β was increased initially and then decreased with the rising TG levels, consisting with the symptoms of dysfunction of β cells.

The glucotoxicity generated by hyperglycaemia is commonly thought to be the fundamental acquired factor causing continuous decline of β cell function in T2DM [2,26]. Sustained hyperglycaemia reduces the β cell function through several ways, such as the increase of oxidative stress, activation of c-Jun N-terminal kinase pathway through activated p38 mitogen-activated protein kinase and protein kinase C, the reduction of the pancreatic and duodenal homeoboxfactor-1 function, and the reduction of ERp46 expression [27-29]. A previous study[30] involving young adults (n = 223) with FPG < 126 mg/dL who underwent an evaluation of first and second phase insulin secretion during a 2 h hyperglycemic clamp found that the impairment in β cell function relative to insulin sensitivity was apparent even within the FPG range of those without diabetes; at the cutoff of FPG 100 mg/dL, there was an approximately 49% decline in the DI. In agreement, we observed that the patients with higher levels of TG had higher levels of TC, FPG, 2hPG, and HbA1c and significantly reduced levels of HOMA-β than other three groups. However, we did not observe significant differences in IGI and DI levels among all four groups of patients. This discrepancy may be due to differences in races of the patients and the limitations of study design.

Several studies have shown that the significance of insulin resistance and insulin secretion defect to diabetes development is linked to patients’ race. For example, the increased insulin resistance to diabetes development is a more prominent factor in Pima Indians, Mexican Americans and Caucasians; whereas the contribution of impaired insulin secretion is a more important factor in Japanese patients [31-33]. It is also reported that the β cell capacity is relatively lower in Asian than that in Western individuals [34-37]. Ma et al. [23] indicated that the Chinese patients of newly diagnosed T2DM with hyperlipidemia were younger and had declined HOMA-β; however, there were no differences in HOMA-IR and quantitative insulin sensitivity check index. Qian et al. [38] indicated that the early diabetes was more specifically characterized by declined insulin secretion rather than impaired insulin sensitivity. In the present study, though the insulin concentration curves indicate that patients with high TG level had a very obvious insulin resistance, a more detailed analysis with additional divisions of TG levels showed that the β cell function increases with the rising TG levels at the early phase, but later decreases as the TG levels further rise. Insulin resistance doesn’t deteriorate as the TG levels further rise. Taken together, therefore, we propose that insulin secretion dysfunction, rather than insulin resistance, may play an important role in the progression of T2DM in Chinese patients.

## Conclusions

Our data indicate that hypertriglyceridemia influences clinical characteristics and β cell function in Chinese patients with newly diagnosed T2DM. A better management of dyslipidemia may, to some extent, reduce the effect of lipotoxicity to diabetes, whereby improving glucose homeostasis in patients with newly diagnosed T2DM. STROBE Statement has been included as an Additional file 1.

## Additional file

Additional file 1:
**STROBE Statement—checklist of items that should be included in reports of observational studies.**
