# Supplementary material for: Clinical characteristics and beta cell function in Chinese patients with newly diagnosed type 2 diabetes mellitus with different levels of serum triglyceride
Source: BMC Endocr Disord. 2015 Apr 29;15:21. doi: 10.1186/s12902-015-0018-1 (PMC4423127; doi:10.1186/s12902-015-0018-1)
Supplement: Additional file 1: — STROBE Statement—checklist of items that should be included in reports of observational studies. [file 12902_2015_18_MOESM1_ESM.docx]

STROBE Statement—checklist of items that should be included in reports of observational studies

|  | Item No. | Recommendation | Page  No. | Relevant text from manuscript |
| --- | --- | --- | --- | --- |
| **Title and abstract** | 1 | (*a*) Indicate the study’s design with a commonly used term in the title or the abstract | 1,2 | Patients with newly diagnosed T2DM (n=624) were enrolled and divided into different groups according to the levels of serum TG |
|  |  | (*b*) Provide in the abstract an informative and balanced summary of what was done and what was found | 1,2 | Hypertriglyceridemia may influence clinical characteristics and β cell function of Chinese patients with newly diagnosed T2DM |
| Introduction | | | |  |
| Background/rationale | 2 | Explain the scientific background and rationale for the investigation being reported | 2,3 | Insulin resistance and impaired insulin secretion are considered as primary pathophysiological factors in the development of T2DM  however, only few studies have focused on the relationship between TG and newly diagnosed T2DM |
| Objectives | 3 | State specific objectives, including any prespecified hypotheses | 3 | In the present study, the clinical characteristics and β cells function in patients with newly diagnosed drug naive T2DM with different levels of TG would be examined. We suppose that newly diagnosed T2DM patients with higher serum TG level might have worse clinical characteristics and more deteriorated β cells function than patients with normal TG level. |
| Methods | | | |  |
| Study design | 4 | Present key elements of study design early in the paper | 3,4 | The patients were firstly divided into two groups according to the serum TG（Group 1: newly diagnosed T2DM with TG of 0 to 1.70mmol/L, n=348; Group 2: newly diagnosed T2DM with TG over 1.70mmol/L, n=276）and then divided into four groups according to the quartile of serum TG (Group 1: newly diagnosed T2DM with TG of 0 to 1.13mmol/L, n=152; Group 2: newly diagnosed T2DM with TG of 1.14 to 1.56mmol/L, n=158; Group 3: newly diagnosed T2DM with TG of 1.57 to 2.27mmol/L, n=158; and Group 4: newly diagnosed T2DM with TG of 2.28 to 11.65mmol/L, n=156) for further examination of β cell function. |
| Setting | 5 | Describe the setting, locations, and relevant dates, including periods of recruitment, exposure, follow-up, and data collection | 3,4 | Patients with newly diagnosed T2DM (n=624) were enrolled in the study between January 2008 and December 2009 in Renji Hospital, School of Medicine, Shanghai Jiaotong University, Shanghai, China. |
| Participants | 6 | (*a*) *Cohort study*—Give the eligibility criteria, and the sources and methods of selection of participants. Describe methods of follow-up  *Case-control study*—Give the eligibility criteria, and the sources and methods of case ascertainment and control selection. Give the rationale for the choice of cases and controls  *Cross-sectional study*—Give the eligibility criteria, and the sources and methods of selection of participants | 3 | All patients had been diagnosed with T2DM within 5 months before the study enrollment and all of them were not treated for T2DM. The diagnosis of T2DM was based on WHO diagnostic criteria established in 1998 |
|  |  | (*b*) *Cohort study*—For matched studies, give matching criteria and number of exposed and unexposed  *Case-control study*—For matched studies, give matching criteria and the number of controls per case |  |  |
| Variables | 7 | Clearly define all outcomes, exposures, predictors, potential confounders, and effect modifiers. Give diagnostic criteria, if applicable | 4 | The patients demographic data and clinical data were collected including age, sex, body height, weight, waist circumference (WC), systolic blood pressure (SBP), diastolic blood pressure (DBP), and levels of TG, TC, HDL-C, low density lipoprotein-cholesterol (LDL-C), fasting plasma glucose (FPG), 2h postprandial glucose (2hPG), serum insulin concentrations at different time point, and hemoglobin A1c (HbA1c). |
| Data sources/ measurement | 8* | For each variable of interest, give sources of data and details of methods of assessment (measurement). Describe comparability of assessment methods if there is more than one group | *4* | Blood pressure was measured after 30 minutes of rest. The body mass index (BMI) was calculated as weight (kg) divided by the square of height (m^2^). Plasma lipid profiles were determined using fully automatic biochemistry analyzer (Hitachi 7020, Hitachi Co., Tokyo, Japan). A standard oral glucose tolerance test (75g glucose load) and insulin releasing tests were performed after a 10-hour overnight fast. Plasma samples were obtained at 0, 30, 60, 120, and 180 minutes to measure glucose (Hitachi 7600-110, Hitachi Co., Tokyo, Japan) and insulin (immunoradiometric assay kit, Dainabot, Tokyo, Japan) concentrations. The HbA1c levels were measured using a high-performance liquid chromatography. |
| Bias | 9 | Describe any efforts to address potential sources of bias |  |  |
| Study size | 10 | Explain how the study size was arrived at |  |  |

Continued on next page

| Quantitative variables | 11 | Explain how quantitative variables were handled in the analyses. If applicable, describe which groupings were chosen and why | 4 | Normality was tested using the one-sample Kolmogorov-Smirnov criterion. Non-normally distributed data were log transformed before analysis. Data were expressed as mean ± standard deviation for normally distributed variables and as median (Interquartile range was 25-75%) |
| --- | --- | --- | --- | --- |
| Statistical methods | 12 | (*a*) Describe all statistical methods, including those used to control for confounding | 4,5 | Independent-samples t test and one-way analysis of variance test were used for normality distributed data. Mann-Whitney U test and Kruskal-Wallis H test were used for non-normal distributed data and alpha level was adjusted to reduce the error risk. |
|  |  | (*b*) Describe any methods used to examine subgroups and interactions | 4,5 | Independent-samples t test and one-way analysis of variance test were used for normality distributed data. Mann-Whitney U test and Kruskal-Wallis H test were used for non-normal distributed data and alpha level was adjusted to reduce the error risk. |
|  |  | (*c*) Explain how missing data were addressed |  |  |
|  |  | (*d*) *Cohort study*—If applicable, explain how loss to follow-up was addressed  *Case-control study*—If applicable, explain how matching of cases and controls was addressed  *Cross-sectional study*—If applicable, describe analytical methods taking account of sampling strategy |  |  |
|  |  | (*e*) Describe any sensitivity analyses | 4 | A P value of <0.05 was considered statistically significant. |
| Results | | | | |
| Participants | 13* | (a) Report numbers of individuals at each stage of study—eg numbers potentially eligible, examined for eligibility, confirmed eligible, included in the study, completing follow-up, and analysed | 5 | Patients with newly diagnosed T2DM (n=624) were enrolled in the study |
|  |  | (b) Give reasons for non-participation at each stage |  |  |
|  |  | (c) Consider use of a flow diagram |  |  |
| Descriptive data | 14* | (a) Give characteristics of study participants (eg demographic, clinical, social) and information on exposures and potential confounders | 5 | Table1 |
|  |  | (b) Indicate number of participants with missing data for each variable of interest |  |  |
|  |  | (c) *Cohort study*—Summarise follow-up time (eg, average and total amount) |  |  |
| Outcome data | 15* | *Cohort study*—Report numbers of outcome events or summary measures over time |  |  |
|  |  | *Case-control study—*Report numbers in each exposure category, or summary measures of exposure |  |  |
|  |  | *Cross-sectional study—*Report numbers of outcome events or summary measures | *5* | *624* |
| Main results | 16 | (*a*) Give unadjusted estimates and, if applicable, confounder-adjusted estimates and their precision (eg, 95% confidence interval). Make clear which confounders were adjusted for and why they were included | 5 | Table1 |
|  |  | (*b*) Report category boundaries when continuous variables were categorized | 5 | Table1 |
|  |  | (*c*) If relevant, consider translating estimates of relative risk into absolute risk for a meaningful time period |  |  |

Continued on next page

| Other analyses | 17 | Report other analyses done—eg analyses of subgroups and interactions, and sensitivity analyses |  |  |
| --- | --- | --- | --- | --- |
| Discussion | | | | |
| Key results | 18 | Summarise key results with reference to study objectives | 6,7 | Patients with T2DM are more common to have lipid disorders. In the present study, it was showed that patients of newly diagnosed T2DM with higher level of TG were younger and fatter and they had a worse lipid profiles and glucose profiles than patients with normal TG levels. Moreover, basal β cell function was initially increased along with the rising TG levels and then decreased. Insulin sensitivity was relatively high with low level of TG and then decreased with higher level of TG, but showed no deterioration with the rising of TG levels. |
| Limitations | 19 | Discuss limitations of the study, taking into account sources of potential bias or imprecision. Discuss both direction and magnitude of any potential bias |  |  |
| Interpretation | 20 | Give a cautious overall interpretation of results considering objectives, limitations, multiplicity of analyses, results from similar studies, and other relevant evidence | 9 | Hypertriglyceridemia may influence clinical characteristics and β cell function of Chinese patients with newly diagnosed T2DM. |
| Generalisability | 21 | Discuss the generalisability (external validity) of the study results | 7 | The present study findings were consistent with previous reports. |
| Other information | |  | | |
| Funding | 22 | Give the source of funding and the role of the funders for the present study and, if applicable, for the original study on which the present article is based | 9,10 | This study was supported by the National Scientific Foundation of China (No. 81270946, 81170758, 30670988). |

*Give information separately for cases and controls in case-control studies and, if applicable, for exposed and unexposed groups in cohort and cross-sectional studies.

**Note:** An Explanation and Elaboration article discusses each checklist item and gives methodological background and published examples of transparent reporting. The STROBE checklist is best used in conjunction with this article (freely available on the Web sites of PLoS Medicine at http://www.plosmedicine.org/, Annals of Internal Medicine at http://www.annals.org/, and Epidemiology at http://www.epidem.com/). Information on the STROBE Initiative is available at www.strobe-statement.org.
